# Time-multiplexed two-channel capacitive radiofrequency hyperthermia with nanoparticle mediation

**DOI:** 10.1186/s12938-015-0090-9

**Published:** 2015-10-24

**Authors:** Ki Soo Kim, Daniel Hernandez, Soo Yeol Lee

**Affiliations:** Department of Biomedical Engineering, Kyung Hee University, Yongin-si, Gyeonggi 446-701 Korea

**Keywords:** Capacitive RF hyperthermia, Time-multiplexed RF heating, Nanoparticles, FDTD simulation, MR thermometry

## Abstract

**Background:**

Capacitive radiofrequency (RF) hyperthermia suffers from excessive temperature rise near the electrodes and poorly localized heat transfer to the deep-seated tumor region even though it is known to have potential to cure ill-conditioned tumors. To better localize heat transfer to the deep-seated target region in which electrical conductivity is elevated by nanoparticle mediation, two-channel capacitive RF heating has been tried on a phantom.

**Methods:**

We made a tissue-mimicking phantom consisting of two compartments, a tumor-tissue-mimicking insert against uniform background agarose. The tumor-tissue-mimicking insert was made to have higher electrical conductivity than the normal-tissue-mimicking background by applying magnetic nanoparticle suspension to the insert. Two electrode pairs were attached on the phantom surface by equal-angle separation to apply RF electric field to the phantom. To better localize heat transfer to the tumor-tissue-mimicking insert, RF power with a frequency of 26 MHz was delivered to the two channels in a time-multiplexed way. To monitor the temperature rise inside the phantom, MR thermometry was performed at a 3T MRI intermittently during the RF heating. Finite-difference-time-domain (FDTD) electromagnetic and thermal simulations on the phantom model were also performed to verify the experimental results.

**Results:**

As compared to the one-channel RF heating, the two-channel RF heating with time-multiplexed driving improved the spatial localization of heat transfer to the tumor-tissue-mimicking region in both the simulation and experiment. The two-channel RF heating also reduced the temperature rise near the electrodes significantly.

**Conclusions:**

Time-multiplexed two-channel capacitive RF heating has the capability to better localize heat transfer to the nanoparticle-mediated tumor region which has higher electrical conductivity than the background normal tissues.

## Background

Capacitive radiofrequency (RF) hyperthermia is one of the hyperthermia methods that aim to selectively induce cancer cell death by delivering heat to the cancer tissues [1, 2]. It is well recognized that cancer cells are more prone to death by heat than normal cells due to limited blood supply to the cancer tissues during heating [3]. Capacitive RF hyperthermia systems, mostly operated at the frequency of 13.56 MHz because of its public availability for general application, have simple configurations. A typical capacitive RF hyperthermia system consists of a surface electrode pair placed at opposite sides of the tumor region and a RF power system to apply RF potential to the electrodes [4]. RF potential difference produces electric field between the electrode and the electric field induces Joule heating in the tissues. Higher Joule heating in the tumor tissues than in the background tissues are highly desired, and higher electric conductivity of the tumor tissues contributes to higher heat transfer to them to some extent. However, since the electrodes are directly contacted to the patient’s skin, higher current density and hence higher heat transfer inevitably appear at the contact region. Even though the electrode has a cooling layer, skin burning may happen nearby the electrode, which may complicate the RF hyperthermia treatment.

In addition to skin burning, there is another big problem in capacitive RF hyperthermia, that is, poorly localized heat transfer. Unlike ultrasound or microwave hyperthermia in which an array of transducers or antennae are employed to focus the energy delivery to the tumor region, capacitive RF hyperthermia only relies on higher electric conductivity of tumor tissues to draw more electric currents to the tumor tissues than to the surrounding normal tissues [3]. However, the conductivity difference between the normal and tumor tissues are not so big as to draw substantially higher electric currents to the tumor region. To increase electric current density at the tumor region, nanoparticles coupled with salts may be employed [5–9]. There have been a few reports that nanoparticles coupled with salts can increase the electric conductivity at the nanoparticle-populated region [10, 11].

In this paper, a new method is introduced to better localize heat transfer to the tumor region with reducing temperature rise near the electrodes in capacitive RF hyperthermia. By employing a cooling layer at the electrode, the skin temperature may be maintained at a desired level during the RF hyperthermia, but, fatty tissues below the skin may not be sufficiently cooled down due to the low thermal conductivity of fatty tissues. To overcome these problems, two electrode pairs, which are placed perpendicularly to one another, are employed in this study. The RF power is then delivered to the electrode pairs in a time-multiplexed way so that only one electrode pair is operating at a time. To monitor the temperature rise all over the interested region, magnetic resonance (MR) thermometry is employed in this study. MR thermometry is based on the linear response of the proton resonance frequency (PRF) of water molecules to the temperature [12–15]. Finite-difference-time-domain (FDTD) simulations on a phantom model, consisting of tumor-tissue-mimicking and normal-tissue-mimicking compartments, has been performed to verify the RF heating experiment inside a 3T MRI magnet with monitoring the temperature inside the phantom. The experimental temperature rise in the phantom is compared with the temperature rise in the FDTD simulation model.

## Methods

### The tissue-mimicking phantom

**Fig. 1:**
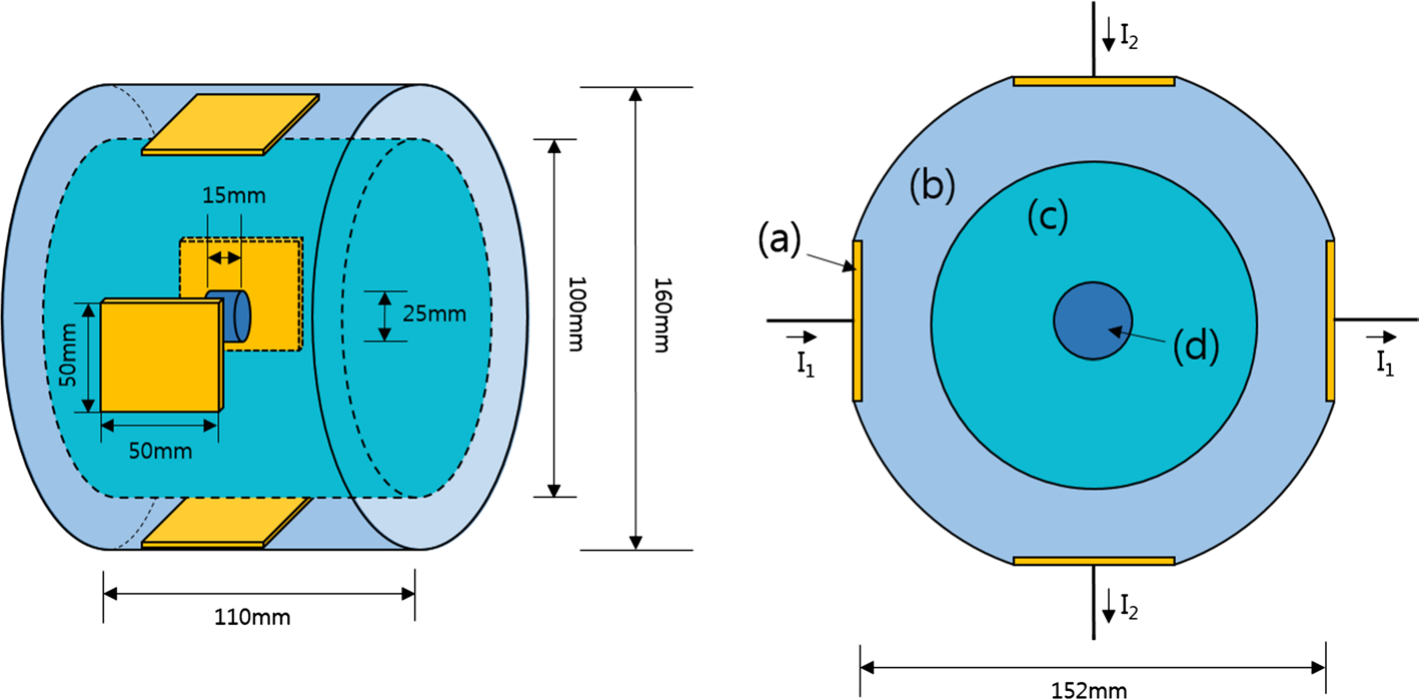
A schematic diagram of the phantom. It consists of four copper electrodes *a*, a distilled water layer *b*, a normal-tissue-mimicking main body *c* and a tumor-tissue-mimicking insert *d*. I_1_ and I_2_ are the RF currents at channel 1 and channel 2, respectively

Figure 1 shows a schematic diagram of the phantom for both the FDTD simulation and the capacitive RF heating experiment. The phantom consists of a tumor-tissue-mimicking insert immersed in the middle of the normal-tissue-mimicking background agarose, a distilled water layer for seamless electric contact with the electrodes, and four copper electrodes. The distilled water layer, with the thickness of 26 mm, also acts as a cooling layer to some extent even though no active cooling device is employed at the experiment. Since the electric conductivity of the distilled water is very low, there is little Joule heating at the distilled water layer, and so, it can absorb heat from the surface of the phantom body. The tumor-tissue-mimicking insert has direct contact with the background agarose without any separating layers between them. The copper electrodes have the size of 50 × 50 mm and they are placed at the outer rim of the distilled layer. The opposing electrode pairs constitute horizontal and vertical channels for RF heating experiment. The electric currents in the horizontal and vertical directions are denoted as I_1_ and I_2_, respectively, in Fig. 1.

The normal-tissue-mimicking compartment, the diameter of 100 mm and the height of 110 mm, was made of 2.5 % agarose (Yakuri Pure Chemicals Co., Japan) and 0.125 % CuSO_4_ solution. CuSO_4_ ionic solution was mixed to control both electrical conductivity and spin–lattice relaxation time (T_1_) of agar. CuSO_4_ shortens T_1_ of water molecules which can facilitate fast magnetic resonance imaging. The concentration of CuSO_4_ was determined by a trial-and-error approach to make electrical conductivity be around 0.1 S/m which is similar to the electrical conductivity of muscular tissues. The tumor-tissue-mimicking insert, the diameter of 25 mm and the height of 25 mm, was made of 2.5 % agarose, 0.125 % CuSO_4_, 4 % carboxymethyl cellulose (Sigma Aldrich, USA) and 0.4 mmol/L Fe_3_O_4_ nanoparticle suspension (Magnetite, RND Korea, Korea). The carboxymethyl cellulose (CMC) and magnetic nanoparticle suspension were mixed with the agarose gel at 60 °C with slow whirling for 40 min, and then, the mixed agarose was cooled down slowly for solidification. The average diameter of the magnetic nanoparticles was 17.5 nm.

Electric permittivity and conductivity of the agaroses were measured by a coaxial surface probe (DAK-12, SPEAG, Switzerland) at the temperature of 25 °C and at the frequency of 26 MHz. The frequency range of the coaxial surface probe was from 10 MHz to 3 GHz. The electric conductivity σ of the normal-tissue-mimicking and tumor-tissue-mimicking agaroses are 0.1 and 0.6 S/m, respectively, with the electric permittivity ε_r_ of 79 and 80, respectively.

### Two-channel RF driving system for RF heating

**Fig. 2:**
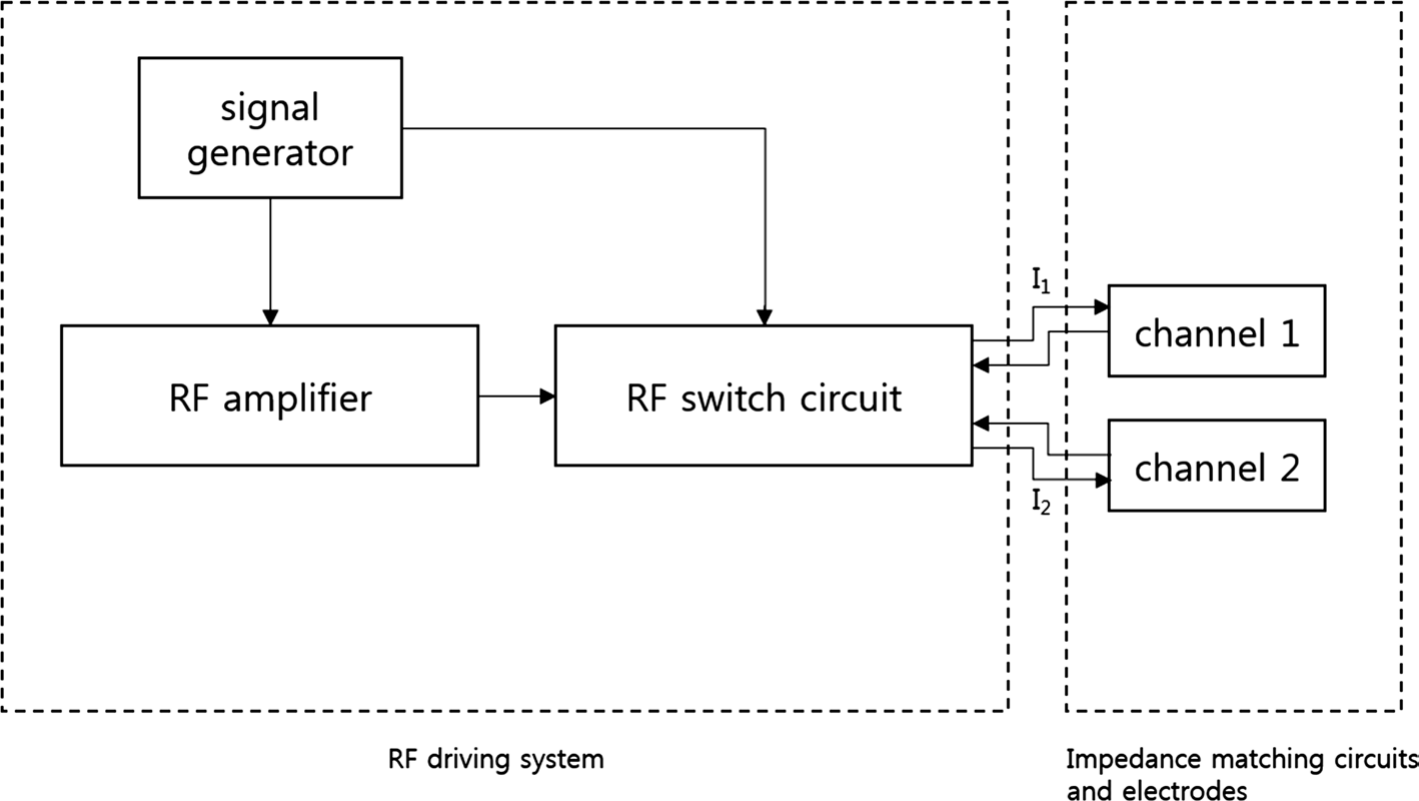
A schematic diagram of the two-channel RF driving system

**Fig. 3:**
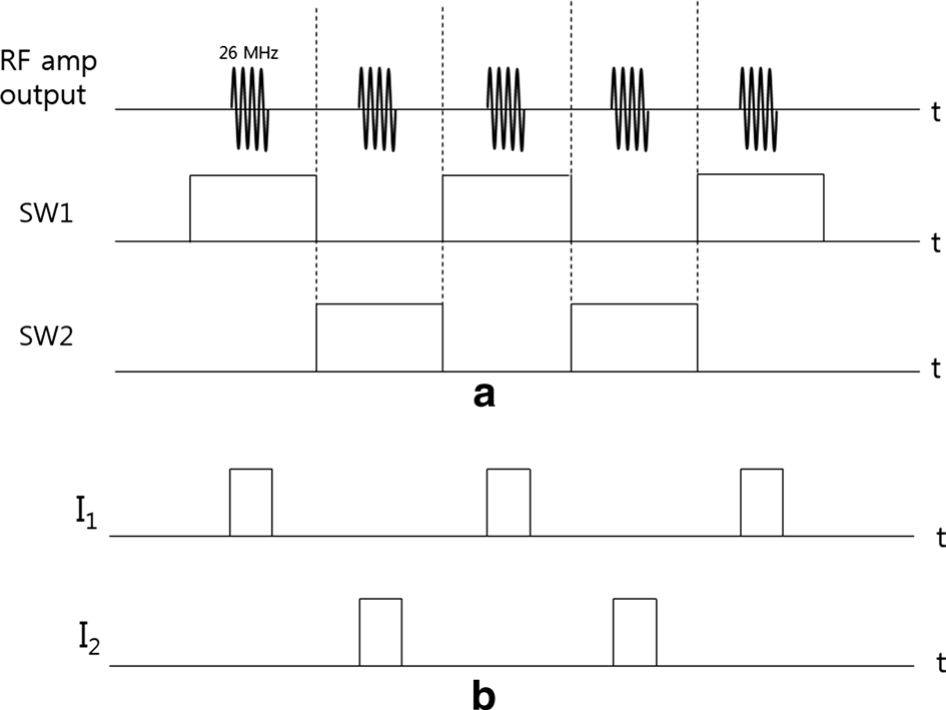
A timing diagram of the alternating RF power delivery to the two channels. **a** RF amplifier output currents and the switching signals. **b** The RF current at each channel

**Fig. 4:**
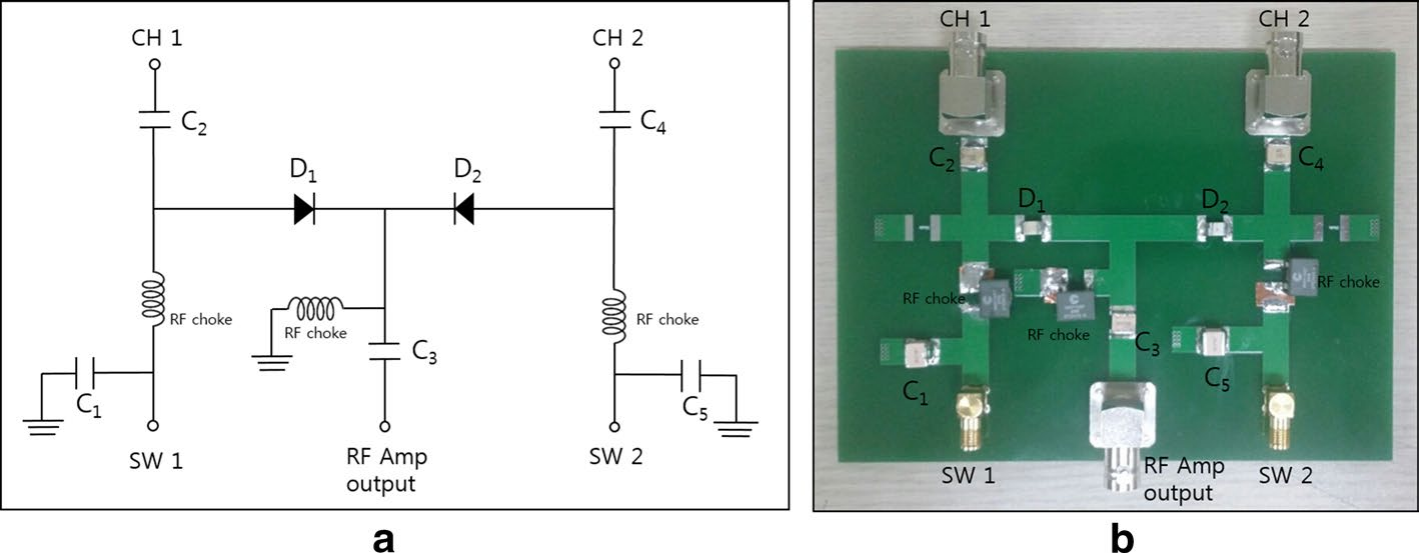
The RF switching circuit for two-channel driving. **a** A circuit diagram and **b** a photography of the RF switching circuit

RF electric potential is applied to the two electrode pairs in a time-multiplexed way. When one electrode pair is driven, the other electrode pair is electrically isolated so that the RF current flowing through one channel does not leak into the other channel. Figure 2 shows a schematic diagram of the two channel driving system with I_1_ and I_2_ indicating the RF current in each channel. The signal generator generates the sinusoidal waveform of 26 MHz to be fed to the RF power amplifier (AMT 3445, Herley, UK) which has the peak power of 2 kW when driven in a pulse mode with the duty cycle less than 20 %. The sinusoidal waveform is generated in a pulsed form with the pulse frequency of 200 Hz and a duty cycle of 9 %. The signal generator also generates the switching signals, SW1 and SW2, to activate the channels in an alternating fashion. After switching, each channel is driven by the RF power amplifier in a pulsed mode with the halved pulse frequency of 100 Hz. Figure 3 shows the timing diagram of the RF pulse waveform and the switching signals. The switching signals are alternating to each other so that the two channels are driven in a time-multiplexed way. Figure 4 shows a circuit diagram and a photography of the RF switching circuit that comprises PIN diodes (DH80106-44 N, Cobham, UK), RF chokes and capacitors. If SW1 is ON, the PIN diode D_1_ is turned on and a RF path from the RF power amplifier to channel 1 is open through the coupling capacitors C_2_ and C_3_, and vice versa D_2_, C_3_, and C_4_ for SW2. RF chokes give ways to DC bias currents to turn on the PIN diodes with blocking the RF currents to SW1 and SW2 ports.

**Fig. 5:**
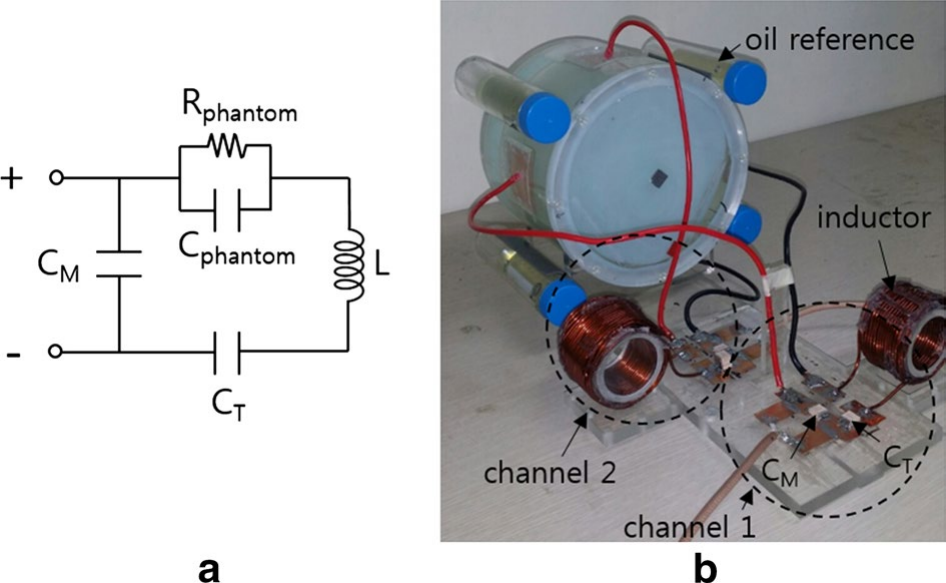
Impedance matching of the phantom circuit. **a** A circuit diagram of the impedance matching circuit and **b** a photography of the impedance matching circuit connected with the phantom. An external inductance L and two external capacitors, C_T_ and C_M_, are connected with the phantom circuit modeled by a parallel circuit of C_phantom_ and R_phantom_

At the RF frequency, the phantom with the electrodes acts as a parallel circuit of a capacitor and a resistor. The capacitance stands for the storage of electric energy in the space between the two electrodes and the resistance stands for the Joule heating by the RF currents. To efficiently deliver RF power to the phantom body without power reflection, the impedance of the phantom circuit has to be matched to the characteristic impedance of the RF system, 50 Ohms in this study. An external inductance L and two capacitors, denoted C_T_ and C_M_, are connected with the electrodes for the impedance matching as shown in Fig. 5. By adjusting capacitance values of C_T_ and C_M_ under the guidance of a network analyzer (Protek A333, GSI, Korea), the impedances of the two channels are matched to 50 Ohms.

### Electromagnetic and thermal simulation

**Fig. 6:**
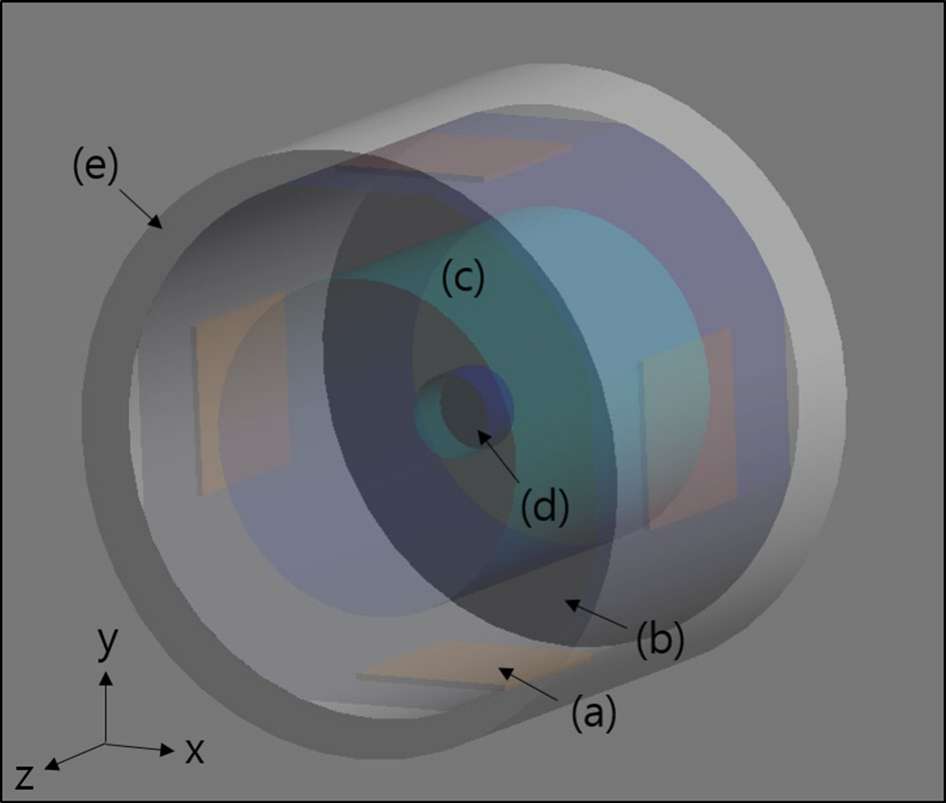
The FDTD model used for the electromagnetic and thermal simulations. It consists of four copper electrodes *a*, a distilled water layer *b*, a normal-tissue-mimicking main body *c*, a tumor-tissue-mimicking insert *d*, and an outer acrylic frame *e*

To validate the experimental results, electromagnetic and thermal simulations have been performed using a finite-difference-time-domain (FDTD) solver (Sim4Life, ZMT, Switzerland) [16]. A FDTD model was built for the phantom with the same physical dimension as the real phantom as shown in Fig. 6. To simulate the two-channel RF heating, RF power of 40 W was applied to one channel with the other channel opened, and vice versa for the other channel. After computing the electromagnetic field produced by each channel, the specific absorption rates (SARs) of the two channels were combined to compute the final SAR in two-channel driving. To simulate the single-channel RF heating, RF power of 80 W was applied to the horizontal channel. In the FDTD electromagnetic simulation, the Maxwell equation was solved with the Yee grid size of 1 mm. The phantom model was placed in the free space, and the uniaxial perfectly matched layers absorbing boundary condition (UPML-ABC) was applied to the outer boundary of the free space. The temperature dependence of the electric properties of the phantom material was ignored.

The SAR distribution computed in the electromagnetic simulation was input to the thermal solver as a heat generator to compute temperature rise inside the phantom. The Pennes bioheat equation was solved with applying Dirichlet boundary condition between the phantom body and the distilled water layer with consideration of cooling effect of the distilled water layer [17]. Dirichlet boundary condition was also applied between the outer acryl frame and the free space. The temperature of the free space was set to 18 °C, the actual ambient temperature of the MRI shield room. It was assumed that the effects of inaccurate boundary condition setting in the FDTD computation be not significant considering the distance from the phantom surface to the region of interest.

Table 1 summarizes the physical parameters of the phantom materials used for the electromagnetic and thermal simulations. Except electrical conductivity, all other parameters were set to the same values for the normal-tissue-mimicking and tumor-tissue-mimicking compartments since main substance of the two compartments were agarose gel with the same composition. The measured values were used for the electrical conductivity and permittivity of the agarose, and other physical quantities were taken from the literatures [18].

**Table 1:** Physical parameters for the electromagnetic and thermal simulations

	Normal-tissue-mimicking agarose	Tumor-tissue-mimicking agarose	Distilled water	Acryl
Conductivity σ (S/m)	0.1	0.6	0	0
Relative permittivity ε_r_	78.78	79.86	76.7	3.0
Physical density ρ (kg/m_)_^3^	1020	1020	1000	1190
Specific heat capacity c_p_ (J/kg/K)	4267	4267	4180	1470
Thermal conductivity k (W/m/K)	0.555	0.555	0.58	0.19

### RF heating experiments with MR thermometry

**Fig. 7:**
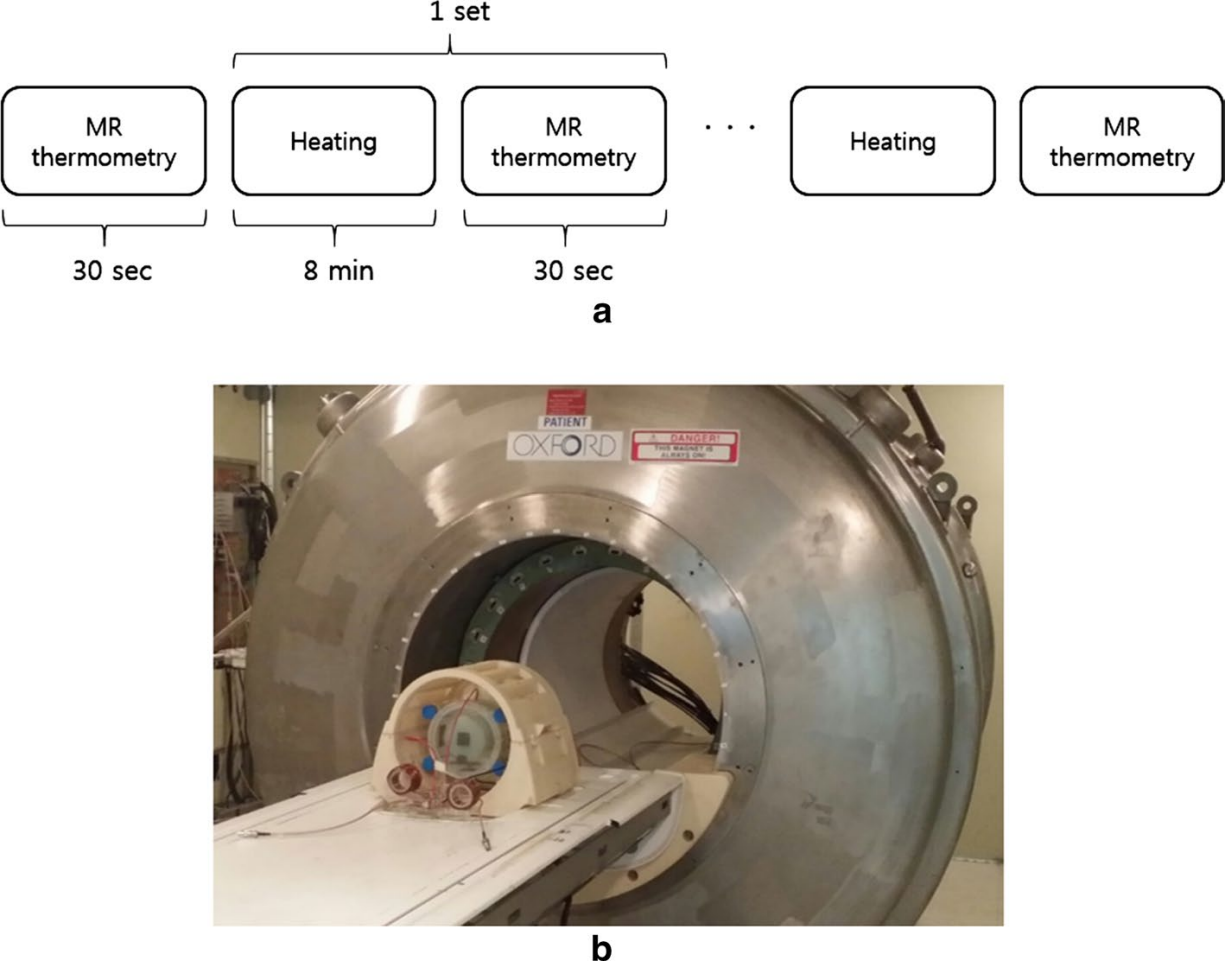
RF heating experiments with intermittent MR thermometry. **a** The timing sequence of the RF heating and MR thermometry experiments. **b** A photography of the phantom placed in the birdcage coil near the 3T MRI magnet

RF heating inside a 3T MRI magnet was performed with intermittent MR thermometry to monitor the temperature rise inside the phantom. As shown in Fig. 7, RF heating and MR thermometry were performed in an alternating fashion with the time frame of 8 min and 30 s, respectively. The phantom was placed inside a birdcage RF coil with the diameter of 28 cm. The birdcage coil was a high-pass type with 16 rungs, and it was used for both RF transmission and signal reception for MRI. The phantom was placed in a way that the central axis of the four electrodes lay on the z-axis, the main magnetic field direction, to minimize the interaction between the birdcage coil and the electrodes. Doing so, the shading artifacts were reduced near the electrodes in the magnetic resonance images of the phantom. The matching circuits were placed outside the birdcage coil to reduce the electromagnetic coupling between the birdcage coil and the matching circuits.

Ten hours before the RF heating experiment, the phantom was placed in the RF coil to make the thermal equilibrium between the phantom and the ambient space in the MRI shield room. The ambient temperature in the MRI shield room was 18 °C. RF heating was repeated three times, so the total heating time was 24 min. MR thermometry was repeated four times with an additional MR thermometry before the RF heating experiment. The first MR thermometry was for making a reference phase map at the initial temperature of 18 °C. RF heating experiments were performed two times, one in the two-channel heating configuration and another in the one-channel heating configuration. In the one-channel heating experiment, the horizontal channel was chosen arbitrarily, and the channel was driven without switching.

For MR thermometry, the proton resonance frequency shift (PRFS) method was used in conjunction with the gradient echo imaging sequence [12]. The PRFS method, which is based on the linear dependency of the proton resonance frequency of water molecules on the temperature, is known to be superior to other methods based on diffusion or T_1_ measurement. For MR thermometry, phase maps are obtained inside the phantom using the gradient echo sequence with echo time TE. The temperature rise ΔT at a given pixel is given by:

1 $$\Delta {\text{T}} = \frac{\Delta \phi }{{\alpha {\text{TE}}\gamma B_{0} }}$$

where α is the thermal coefficient of proton resonance frequency shift (−0.01 ppm/^o^C for protons in water molecule), γ is the gyromagnetic ratio of hydrogen (42.58 MHz/T), *B*_o_ is the main magnetic field strength, TE is the echo time, and Δϕ is the phase shift caused by temperature change. Phase shifts in MR images can be induced by other factors than temperature change. Particularly in MR thermometry with a long scan time, main magnetic field (*B*_o_) drift, caused by the temperature variation in the superconducting magnet, could make big phase shift. The phase shift caused by *B*_o_ drift can be space-variant, so it can compromise the temperature mapping accuracy. Therefore, the phase shift caused by the *B*_o_ drift must be distinguished from the phase shift caused by the temperature change.

To correct the *B*_o_ drift effect on the temperature mapping, four oil tubes with the diameter of 22 mm were placed near the phantom as shown in Fig. 5b. The thermal coefficient of proton resonance frequency of oil molecules is far less than that of water molecules, hence, the phase shift in the oil segments in MR images solely depends on the *B*_o_ drift [19–21]. Therefore, by measuring the phase shift at the oil segments, the amount of *B*_o_ drift can be estimated at the place of the oil tubes. Bilinear interpolation was applied to obtain the phase drift map, Δϕ_Bo_, inside the phantom body from the four phase shift values at the four oil tubes. To mitigate the noise effects, the phase shift value at a given oil tube was computed by taking the average over 5 × 5 pixels in the middle of the oil tube. The temperature change after correcting the *B*_o_ drift effect is now given by:

2 $$\Delta {\text{T}} = \frac{{\Delta \phi - \Delta \phi_{{B_{0} }} }}{{\alpha {\text{TE}}\gamma B_{0} }}$$

For MR thermometry, the gradient echo imaging sequence was used with the repetition time (TR) and echo time (TE) of 110/10 ms, the flip angle of 60^o^, and the image matrix size of 128 × 128 over the field of view of 220 × 220 mm. The number of averages was two which made the scan time for each round of MR thermometry be about 30 s. To set a temperature reference in MR thermometry, an optic fiber temperature sensor was placed in the middle of the phantom and the temperature read by MR thermometry was compared with the one read by the optic fiber sensor.

## Results

**Fig. 8:**
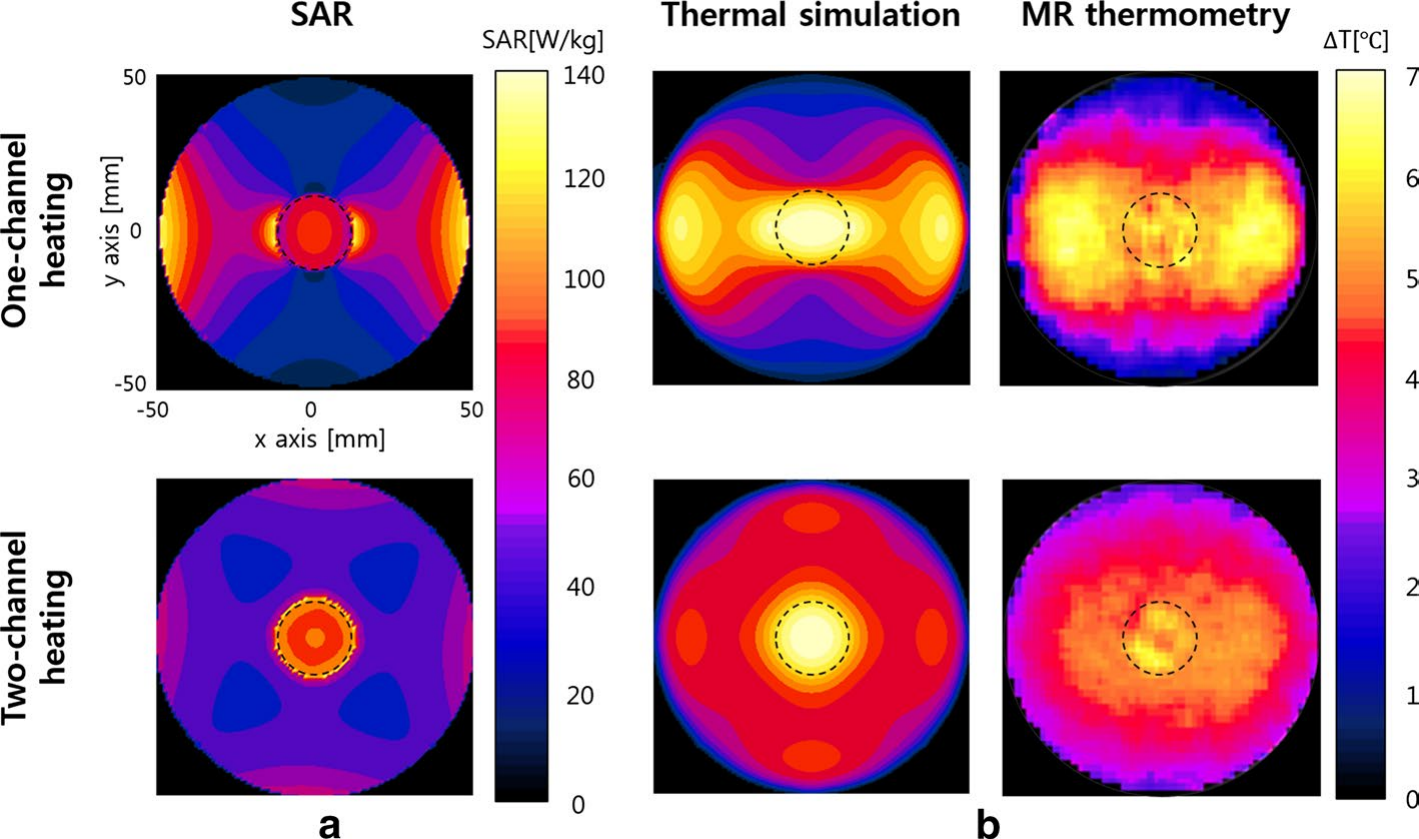
SAR and temperature maps in the simulations and experiments. **a** Simulated SAR maps for the one-channel and two-channel RF heating. **b** Simulated and experimental temperature maps at the time of 24 min RF heating

Figure 8 shows the simulated SAR and temperature maps at the time of 24 min after starting RF heating in the cases of one-channel and two-channel RF heating. From the SAR map of the one-channel RF heating in the horizontal direction, high SAR is noticed near the electrodes, which is caused by high electrical current density near the electrodes. The current near the electrodes spreads out through the phantom, but more currents are drawn in the tumor-tissue-mimicking insert due to its higher electrical conductivity. However, higher SAR appears at both sides of the tumor-tissue-mimicking insert than inside the insert. According to the law of continuity of electric current in the space, the normal component of the current must be continuous at the interface of the insert. Therefore, higher SAR is formed at both sides of the insert due to the higher resistance of the normal-tissue-mimicking background. At the top and bottom of the insert, however, lower SAR appears because the electric currents are mostly tangential to the insert surface. Unlike the normal component, the tangential component of the electric currents may be discontinuous at the interface depending on the conductivity difference between the two regions. Unlike SAR distribution, the temperature rise near the electrodes is not as high as near the insert, which is due to the cooling effect of the distilled water layer. In the two-channel RF heating, both SAR and temperature rise are better localized at the tumor-tissue-mimicking insert, which demonstrates the localizing capability of the time-multiplexed two-channel RF heating. The average temperature rises inside and outside the insert, circled in the temperature map, are 5.5/3.7 °C in the one-channel RF heating and 5.3/3.6 °C in the two-channel RF heating. From the simulation results, it can be seen that the two-channel RF heating gives similar temperature rise in the tumor region but with better localized heat transfer. In the two-channel RF heating, the temperature rise near the electrodes are much reduced because the electric current at one electrode pair is reduced by factor of √2.

**Fig. 9:**
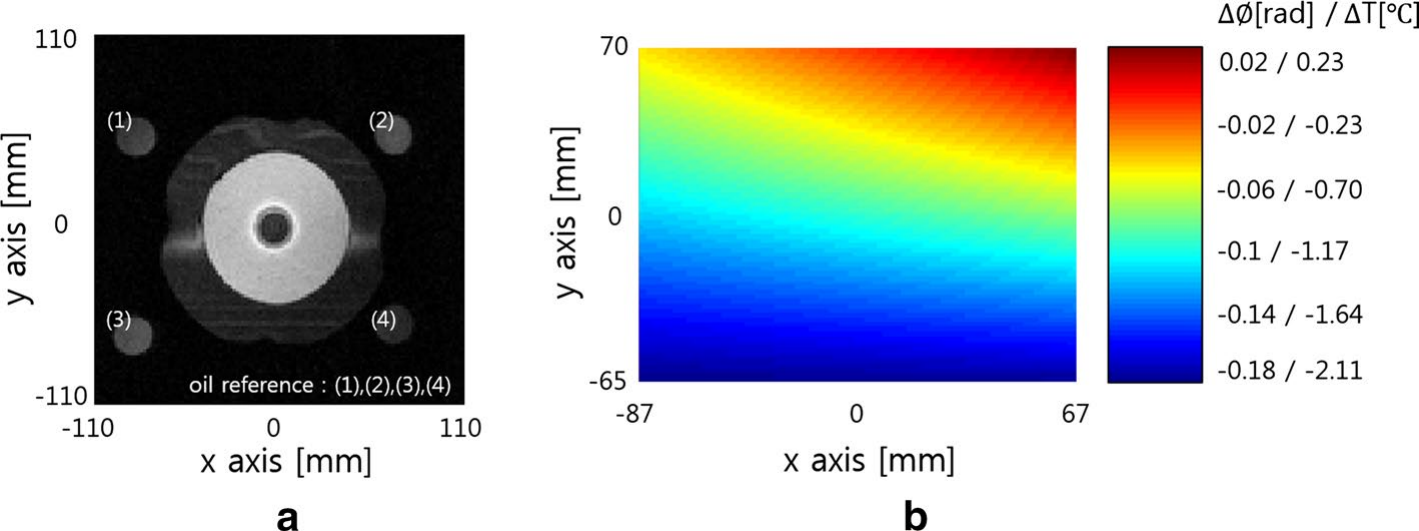
An axial-view image of the phantom and oil tubes and the B_o_-drift-induced phase map. **a** An axial-view MR image of the phantom and four oil tubes. **b** The B_o_-drift-induced phase map computed by applying bilinear interpolation to the phases at the oil tube position, which demonstrates possible temperature errors ranging from −2.11 to 0.23 °C if not corrected

Figure 9a shows a gradient echo image of the phantom and four oil tubes. The phase shift at each oil tube was measured by taking the average of 5 × 5 pixels in the middle of oil tube. The phase map over the square region the size of 135 × 154 mm was obtained by applying bilinear interpolation to the four phase values at the oil tube position. Figure 9b shows the phase map at the time of 16 min RF heating, which represents the phase errors caused by the B_o_ drift. The phase errors directly result in temperature mapping errors ranging from −2.11 to 0.23 °C if not corrected. The phase error maps were computed for each round of MR thermometry, and they were used to correct phase errors in the temperature mapping at the time of 8, 16, and 24 min RF heating.

**Fig. 10:**
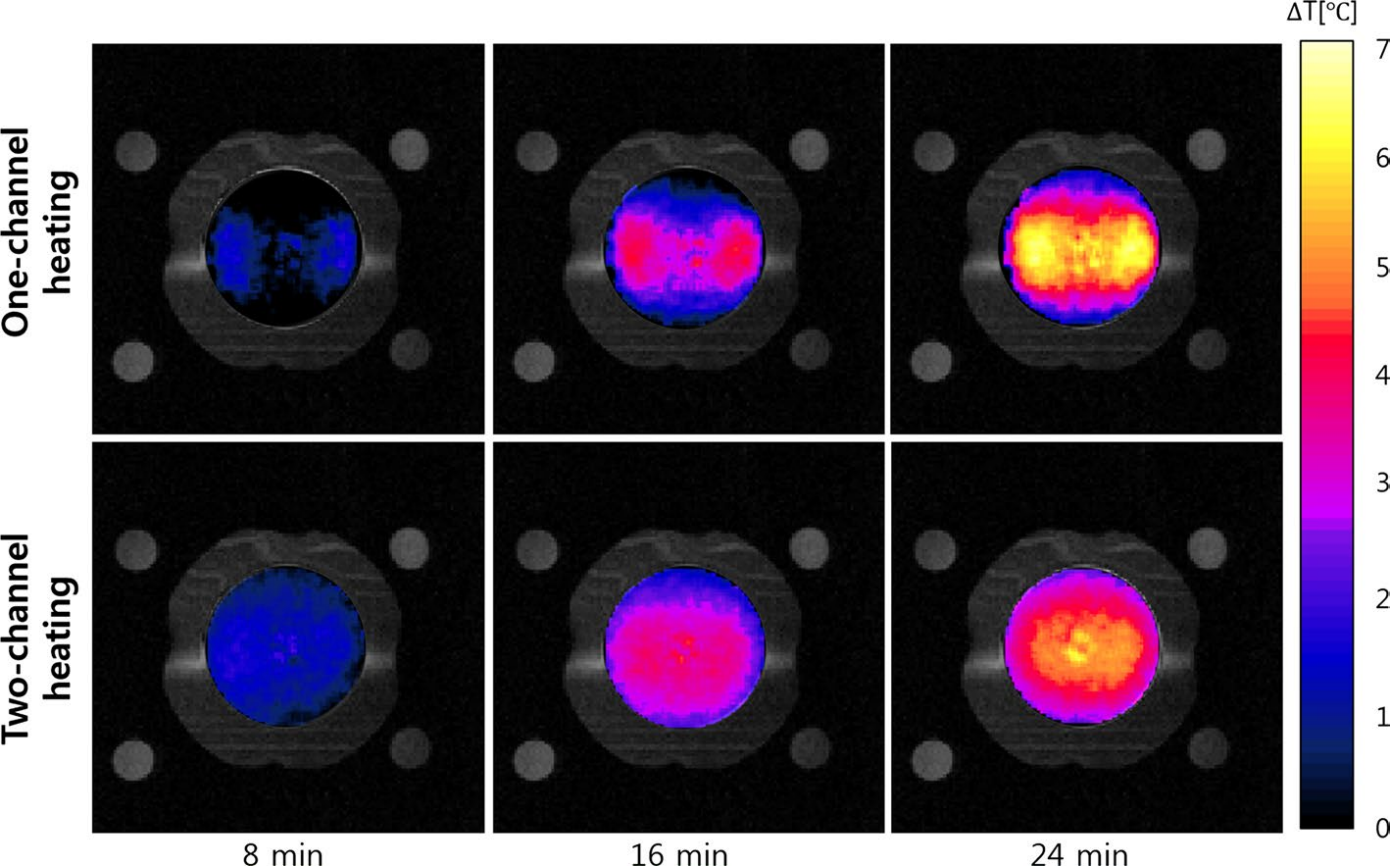
The experimental temperature maps at the time of 8, 16, and 24 min RF heating. The top and bottom rows are the temperature maps for the one-channel and two-channel RF heating cases, respectively

Figure 10 shows the temperature maps inside the phantom body at the time of 8, 16, and 24 min RF heating in the case of one-channel and two-channel RF heating. As RF heating goes on, the temperature inside the phantom continuously rises up to about 7 °C. From the experimental temperature maps too, better localizing is noticed at the tumor-tissue-mimicking insert in the two-channel RF heating. In the one-channel RF heating, however, the region of temperature rise is extended from the tumor-tissue-mimicking insert to the electrodes.

**Fig. 11:**
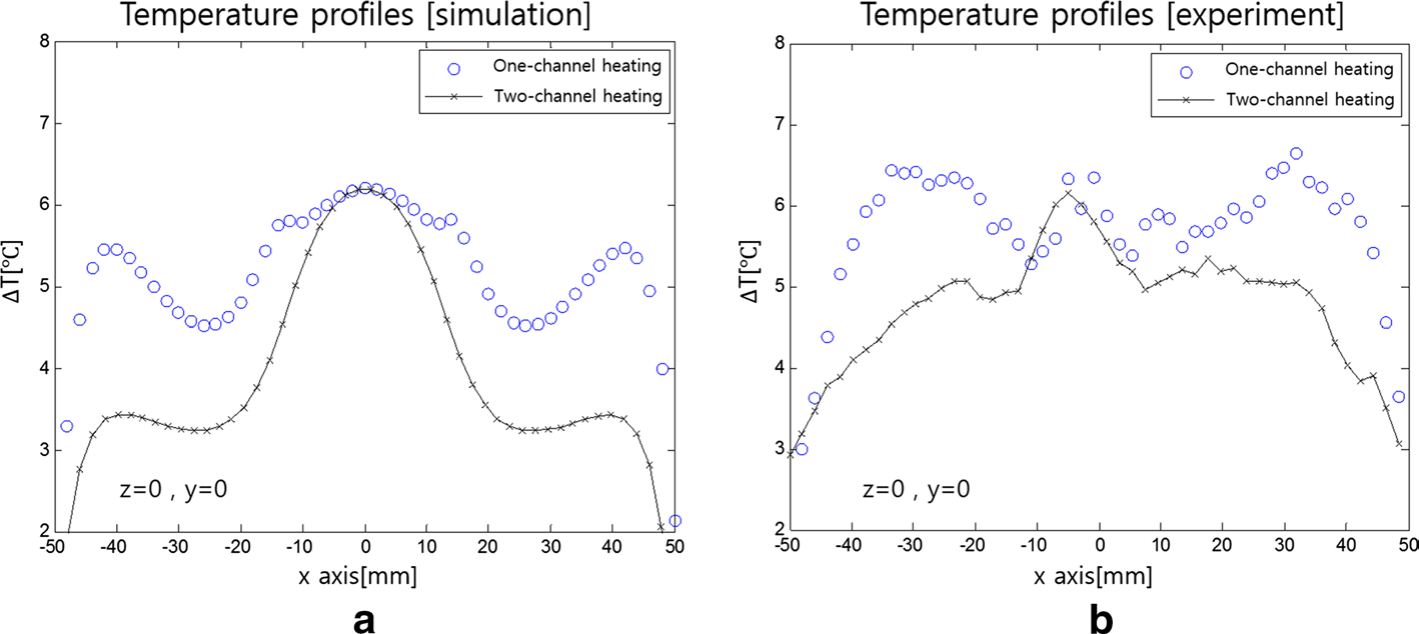
Temperature profiles along the central horizontal line of the phantom. **a** The profiles in the simulation and **b** in the experiment

Figure 11 compares the temperature profiles between the one-channel and two-channel RF heating in the FDTD simulation and experiment. The temperature profiles are taken along the central horizontal line passing through the phantom. In the temperature profiles too, better localized temperature rise is noticed at the tumor-tissue-mimicking insert in both the simulation and experiment.

## Discussion

There were some mismatches of temperature patterns between the experiments and simulations, particularly around the insert as noticed in Figs. 8 and 11. In the physical phantom, a thin PVC film with thickness of 0.2 mm was placed between the insert and the background to prevent molecular diffusion across the interface, and the film may have prohibited thermal conduction across the interface. In the simulation model, the thin film was not considered since the film thickness was too small as compared to the FDTD element size. It is thought that the heating was less localized in actual experiments than simulations due to the thin film.

**Fig. 12:**
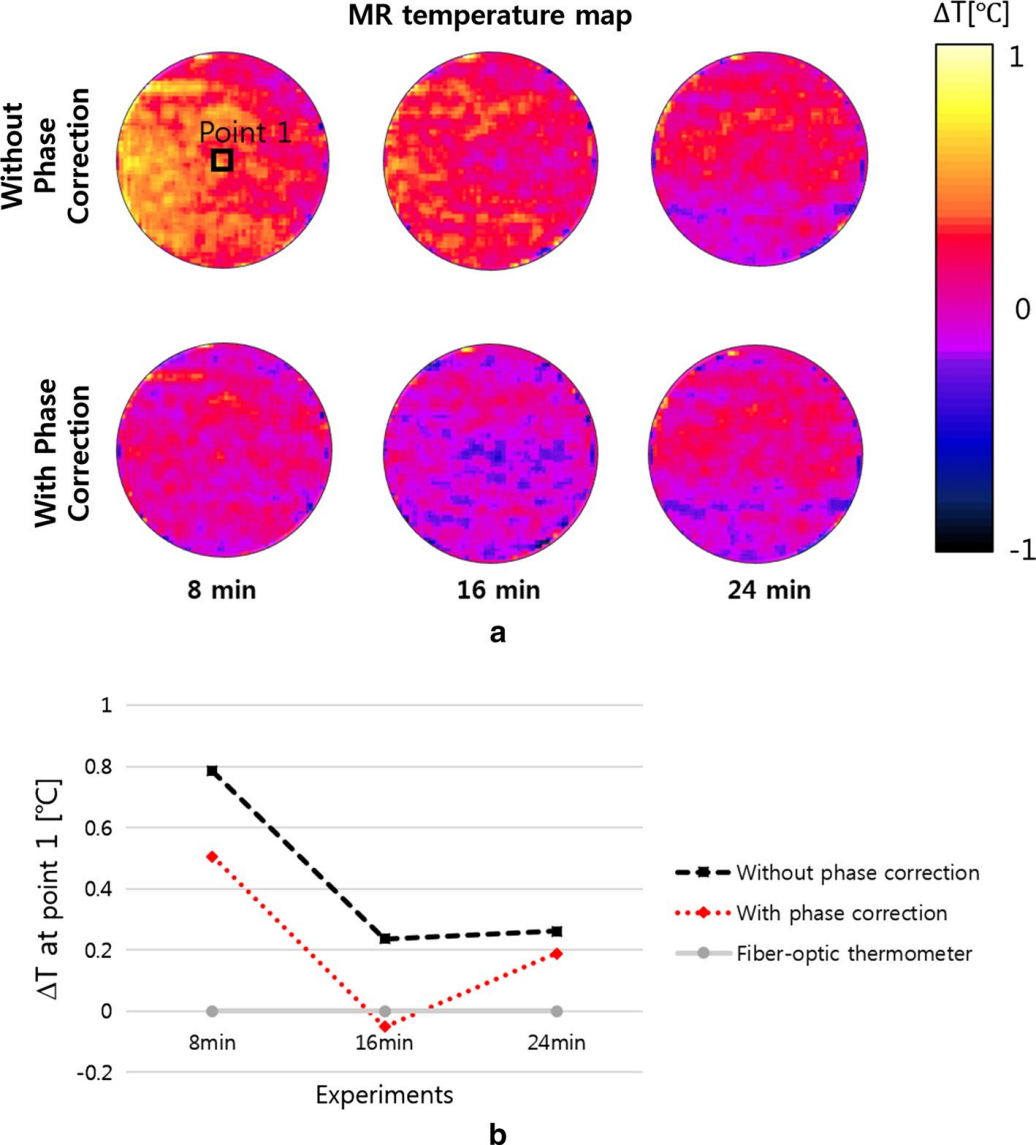
Temperature errors in MR thermometry. **a** The MR temperature maps taken without RF heating at the time of 8, 16, and 24 min. Phase correction reduces spatial temperature fluctuations on the temperature maps. **b** The temperature errors at the center of the phantom between the optic fiber sensor reading and MR thermometry

To evaluate the accuracy of MR thermometry, MR thermometry was performed without RF heating with an optic fiber temperature sensor at the center of the phantom. Without phase error correction, the temperature maps taken at 8, 16 and 24 min show some spatial fluctuations. However, the fluctuations were greatly reduced after phase error correction as shown in Fig. 12a, b shows the temperature errors between the optic fiber measurement and MR thermometry at the center of the phantom. The temperature errors were also significantly reduced after phase error correction.

Multi-channel RF heating in a time-multiplexed way can improve spatial localization of heat transfer to the region of interest. RF currents induced by the RF potential applied to the two electrode pairs are piled up together at the region of interest, which in turn increases SAR at the region. To better localize the heat transfer, the shape and position of the electrodes should be optimized along with consideration of the tumor size, tumor position, and the electrical properties of the tissues. In this study, a simple and symmetric phantom has been used with arbitrary choice of the electrode size and position. If the region of interest is not at the center of the body, and if the electrical conductivity and permittivity are not uniform, the electrode optimization will not be a simple task. However, recent developments in EM simulation on realistic human body models, usually derived from 3D medical images of a human subject, would make it feasible to find optimal shape and position of electrodes for clinical practice.

Spatial localization of heat transfer to the region of interest and reduction of temperature rise at the skin region would be improved if more number of channels are employed. Since RF power amplifier output is divided in a time-multiplexed way, a single RF power amplifier with inexpensive switching circuits would suffice for the multi-channel RF heating with the number of channels greater than two. Multi-channel RF heating also reduces the heat loads at the electrodes which would need higher cooling capacity otherwise.

It is observed that the magnetic nanoparticle suspension mixed with CMC elevates the conductivity of the nanoparticle-populated agarose and the elevated conductivity is maintained for a for a few days. This implies that the nanoparticles somehow bind the ions derived from the CMC salts thereby limiting diffusion of ions to outside the nanoparticle-populated region [22, 23]. There have been a few reports that nanoparticles and salts elevate the heat transfer in capacitive RF heating possibly due to the elevated conductivity at the nanoparticle-populated region [10, 11]. In this study, the nanoparticle-populated region has six times higher conductivity than the background region. Considering that much denser magnetic nanoparticles are injected to the tumor region in human studies of magnetic fluid hyperthermia [24], the conductivity difference in this phantom study, between the nanoparticle-populated region and the background region, would not be unrealistic to emulate the clinical situation. However, the mechanism of conductivity elevation by nanoparticles and salts should be investigated further for the clinical application of nanoparticles in hyperthermia.

MR thermometry was intermittently performed during the RF heating experiment. The phantom was positioned inside the head RF coil and the matching circuits were placed near the coil. With this configuration, little interference was observed between the RF heating devices and the RF coil, and high-quality temperature maps could be obtained. However, for human studies in which large-sized electrodes are employed, coupling between the RF heating devices and the MRI RF system could be so big as to compromise the temperature mapping. Decoupling between them, particularly in high field MRI, would be a technical challenge.

## Conclusions

Two-channel capacitive RF heating in a time-multiplexed way can make better localized heat transfer to the nanoparticle-populated tumor region than one-channel heating. In addition to the better localized heat transfer, the two-channel RF heating can reduce the temperature rise near the surface electrodes. With intermittent MR thermometry during RF heating, the temperature rise can be monitored at the region of interest. It is expected that time-multiplexed multi-channel RF heating would greatly facilitate capacitive RF hyperthermia which suffers from excessive temperature rise near the electrodes and poorly localized heat transfer to the tumor region.
